# Exercise-Induced Angioedema, Urticaria, and Anaphylaxis—A Narrative Review

**DOI:** 10.3390/sports13070215

**Published:** 2025-07-03

**Authors:** Waleed Aman Ur Rahman, Mohamad Mahdi Mortada, Paulina Ślimok, Alaa Sherri, Katarzyna Poznańska-Kurowska, Anna Zalewska-Janowska, Marcin Kurowski

**Affiliations:** 1 Department of Immunology and Allergy, Medical University of Lodz, 90-419 Lodz, Poland; waleed.amanurrahman@student.umed.lodz.pl (W.A.U.R.); mohamadmahdi.mortada1@student.umed.lodz.pl (M.M.M.); 2 GA^2^LEN|HAEi Angioedema Center of Reference and Excellence (ACARE), Departments of Psychodermatology and Immunology and Allergy, Central Teaching Hospital, Medical University of Lodz, 92-213 Lodz, Poland; anna.zalewska-janowska@umed.lodz.pl; 3 Department of Family Medicine, “Salve” Outpatient Clinic, 90-420 Lodz, Poland; 4 Department of Rheumatology, Medical University of Lodz, 90-419 Lodz, Poland; alaa.sherri@umed.lodz.pl; 5 Department of Immune Metabolism, Swiss Institute of Asthma and Allergy Research (SIAF), CH-7265 Davos, Switzerland; 6 Department of Dermatology, Pediatric Dermatology and Oncological Dermatology, Dr. W. Bieganski Regional Hospital, 91-347 Lodz, Poland; k.poznanska@lodz.home.pl; 7 Psychodermatology and Neuroimmunobiology of the Skin Department, Medical University of Lodz, 90-419 Lodz, Poland

**Keywords:** exercise, angioedema, urticaria, acquired angioedema, hereditary angioedema, histaminergic angioedema, bradykinin-mediated angioedema, chronic inducible urticaria

## Abstract

The benefits of regular physical exercise, primarily moderate-intensity exercise, are widely known, recognized, and acclaimed. As an important lifestyle modification, regular training activities are gaining increasing popularity in the general population. Apart from the obvious benefits, physical exercise may carry the risk of trauma, cardiovascular events, and exercise-induced asthma and bronchoconstriction, to name just a few well-known clinical situations reported in athletes, both recreational and competitive. In susceptible individuals, acute bouts of exercise may lead to the appearance of urticaria, angioedema, and anaphylaxis. Among these three clinical phenomena, angioedema is the least addressed and recognized, often being considered an accompanying clinical feature of urticaria or a hallmark of imminent anaphylactic reaction. To fill this knowledge gap, in this review, we focus on exercise-associated angioedema symptoms and highlight their most important features, both as isolated phenomena and in association with urticaria or anaphylaxis.

## 1. Introduction

Angioedema (AE) is a localized, self-limiting swelling reaction with skin and/or mucosal manifestations and sequential to an increase in vascular permeability [1,2,3]. The increase in blood vessel permeability is caused by vasoactive mediators, predominantly bradykinin (BK) and histamine. The role of other mediators, such as prostaglandins, leukotrienes, mast cell tryptase, and various cytokines and chemokines, is likely, but, no conclusive or definite data are available in relation to any of these. The degree of involvement of individual mediators may vary depending on the pathogenesis, circumstances of occurrence, and stimuli causing edema [4]. Localizations of AE vary substantially. Swelling episodes may run a different course, from seemingly benign peripheral localizations at the distant parts of the upper and lower limbs to life-threatening episodes involving pharyngeal and laryngeal mucosa. Abdominal localizations of attacks are also frequent, reported in 43% to 93% patients with hereditary angioedema (HAE) [5,6,7]. In an account of 521 attacks of HAE that occurred in 149 patients, 49% of exacerbations manifested solely with abdominal symptoms [8]. Due to their misleading clinical appearance, gastrointestinal symptoms of HAE may be initially diagnosed as acute abdominal disease (appendicitis, peritonitis) and result in surgical interventions, which indeed can often be ascertained in AE subjects’ medical records [9,10,11,12]. Other clinical features of HAE include the absence of itch and the absence of morphological indices of inflammation at the reaction site. Complexity and variability in the HAE clinical picture are the reason for considerable delay in establishing a diagnosis, reported in particular concerning the hereditary form of angioedema [13].

Regular physical activity is also becoming more common in the general population. At the same time, the incidence of allergic diseases and asthma is on the rise [14,15,16], leading to the fact that allergists, dermatologists, physicians of other specialties, and general practitioners will increasingly have to deal with the consequences of the impact of physical exercise on the development and course of allergy and asthma in their practice. On the other hand, it cannot be neglected, however, that moderate-intensity exercise may contribute to controlling the process of systemic chronic inflammation and confer beneficial effects to general health [17]. AE, although usually being primarily managed by allergists and dermatologists, may result from conditions exceeding the boundaries of these specialties. Not only can it present with a highly variable spectrum of symptoms, as highlighted above, but it also may be accompanied by urticaria and—in extreme cases—be a hallmark of developing life-threatening anaphylactic reaction. Exercise is a stimulus that can activate mechanisms leading to the development of AE, urticaria, or anaphylaxis, either as a sole trigger or acting as a cofactor, amplifying the response to previously identified triggers (e.g., allergen exposure, drug ingestion) or eliciting the response to stimuli previously considered not to have clinical relevance in a given subject [18,19,20].

In this article, we present a review of exercise-associated AE symptoms that may develop as sequelae to physical activity of different types and intensity and highlight their most important features, both as isolated phenomena and in association with urticaria or anaphylaxis.

## 2. Methods

A comprehensive search of Scopus and PubMed databases for articles published until February 2025 was performed. The search query in each database included a combination of the following search terms: “angioedema”, “exercise”, “urticaria”, “anaphylaxis”, “histaminergic angioedema”, and “bradykinin-mediated angioedema.” The inclusion criteria for article selection included relevant original and review articles describing human studies and published in English, without time frame restrictions. After excluding duplicates and articles that did not match the review’s inclusion criteria, 87 articles were included.

## 3. Angioedema: Pathomechanism, Classification, and Clinical Features

AE is a frequent reason for patients to seek medical advice, both in general practice and upon specialist referrals to allergists or immunologists. New classification and terminology of AE syndromes have been developed and were published recently as a joint consensus of the American Academy of Allergy, Asthma & Immunology (AAAAI), American College of Allergy Asthma & Immunology (ACAAI), Angioedema Centers of Reference and Excellence (ACARE), and Asia Pacific Association of Allergy, Asthma and Clinical Immunology (APAAACI) [1]. It provides guidelines for the description and classification of subjects presenting with AE symptoms, dividing AE according to pathogenesis into:

- Mast cell-mediated (AE-MC).
- Bradykinin-mediated (AE-BK).
- AE due to vascular endothelium dysfunction (AE-VE).
- Drug-induced (AE-DI).
- AE of unknown etiology and mechanism.

**Figure 1:**
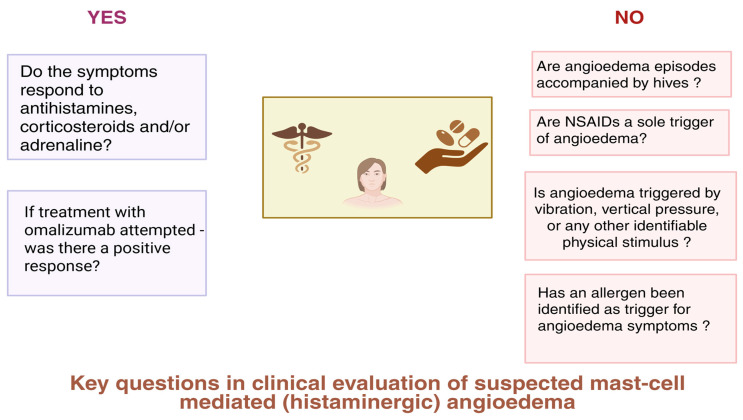
Clinical questions to be considered during evaluation of a patient suspected of having mast cell-mediated angioedema. Created in BioRender. Kurowski, M. (2025) https://BioRender.com/7z458mi (accessed on 25 April 2025).

Differentiating mast cell-mediated and bradykinin-mediated AE is important from the clinical point of view. Initial suspicions concerning possible pathomechanisms are helpful in applying relevant diagnostic tools and prophylactic measures and raising patient awareness about possible triggers [21,22]. During clinical evaluation, several key issues should be addressed while taking a patient’s initial history, which may contribute to prompt skewing of the diagnostic path towards histamine- or bradykinin-mediated AE as a possible diagnosis, depending on prevailing clinical features. As shown in Figure 1, affirmative or negative answers to targeted questions during history-taking may render the diagnosis of mast cell-mediated (histaminergic) AE more probable. In addition, Table 1 presents key features of histaminergic and bradykinin-mediated AE that are recommended for consideration when performing differential diagnosis.

Mast cell-mediated AE (also referred to as histaminergic angioedema (HA)) is considered a subtype of chronic spontaneous urticaria with no concomitant hives [22]. Features such as male sex predominance among affected subjects and absence of IgG antibodies against IgE receptors on basophils or against IgE itself have been ascertained [23], which favor opinions that mast cell-mediated AE should be considered a separate nosology entity and not an urticaria subtype [22].

**Table 1:** Key clinical features of angioedema mediated by histamine and bradykinin. Based on [21,24].

Feature	Histaminergic AE	Bradykinin-Mediated AE
Concomitant skin rash	Urticarial wheals	Usually no urticaria
Timing of symptoms’ onset	Usually rapid	Usually slow
Symptoms’ duration	24–48 h	3–5 days
Family history	Frequently atopic disease	In HAE about 80% patients with family history of recurrent AE and/or confirmed HAE diagnosis
Pruritus	Present	Absent, lesions rather painful than itchy
Typical age of onset	Any	HAE-C1INH: usually childhood or adolescence HAE with normal C1INH: young adults AAE-C1INH and AE-ACEI: after age 40
Submucosal localization—upper airways	May be present, especially if anaphylaxis develops	More frequent
Tongue swelling	Frequent	Frequent in HAE with normal C1INH caused by *PLG* mutations and in AE-ACEI
Gastrointestinal tract involvement	Rare	Present in up to 90% of HAE-C1INH

AE, angioedema; HAE, hereditary angioedema; C1INH, C1-esterase inhibitor; HAE-C1IH, hereditary angioedema due to C1-esterase inhibitor deficiency; AAE-C1INH, acquired angioedema due to C1-esterase inhibitor deficiency; AE-ACEI, angioedema due to angiotensin-converting enzyme inhibitors.

Apart from the classifications presented above, AE may be divided into hereditary (HAE) and acquired (AAE), each associated with different etiological factors.

HAE is a rare genetic disorder with an estimated prevalence of 1:50,000, accounting for up to 2% of cases of all AE syndromes, without significant predilection for sex or ethnicity [9]. Although AE was described as a separate nosology entity for the first time in 1882 by Quincke (referred to as “angioneurotic edema”), with a thorough report indicating the hereditary nature of symptoms provided by Osler in 1888, it was not until 1963 that Donaldson and Evans [25] identified a genetically determined deficiency in the C1-esterase inhibitor (C1INH) as the cause of the recurrent swelling described by Quincke and Osler.

BK is a key player in the pathogenesis of HAE. BK is a vasoactive peptide that contributes to vasodilatation, acting through its receptor BK2. BK is generated after the active plasma kallikrein cleaves high-molecular-weight kininogen (HMWK).

Plasma kallikrein is activated from its inactive zymogen prekallikrein by protease factor XII (FXII), which autoactivates upon contact with negatively charged surfaces. Plasma kallikrein and factor XII are inhibited by C1-INH. Therefore, decreased C1-INH concentration and/or activity contribute to increased BK generation, resulting in increased vascular permeability and swelling [3]. BK is degraded by ACE (angiotensin-converting enzyme), carboxypeptidase N, neutral endopeptidase, dipeptidyl peptidase IV (DPP-IV), and aminopeptidase P [26], hence the possible role of drugs interfering with the function of these proteins in the pathogenesis of an acquired, drug-induced form of AE (see below).

The majority of cases of HAE are due to mutations in the *SERPING1* gene coding the C1INH protein [27,28,29]. The diagnostic approach to these variants of HAE includes plasma measurements of C1INH concentration and activity, as well as C4 complement protein concentration. HAE type 1 is characterized by a decrease in C4, C1INH concentration, and C1INH activity, whereas in HAE type 2, only C1INH activity (along with C4 concentration) is decreased, with C1INH concentration remaining within the normal range [3]. Over the last few years, mutations in genes other than *SERPING1* have been described in subjects with HAE. These newly identified forms of HAE create a considerable diagnostic challenge [30,31,32]. C1INH concentration and activity being within normal ranges, there is no easily accessible laboratory diagnostic test to confirm diagnosis in those cases, and the only way to confirm diagnosis is through genetic testing, preferably NGS (next-generation sequencing) [28,32,33]. Mutations related to HAE with normal C1INH have been described in factor XII (*F12*), plasminogen (*PLG*), angiopoietin 1 (*ANGPT1*), kininogen 1 (*KNG1*), myoferlin (*MYOF*), heparan sulfate (HS)-glucosamine 3-O-sulfotransferase 6 (*HS3ST6*), CPN1 (carboxypeptidase N1) and *DAB2IP* (disabled homologue 2-interacting protein) genes, with several others being studied as potentially associated with the pathogenesis of HAE [34,35,36,37].

Acquired angioedema (AAE) is far more prevalent than HAE, yet equally challenging in terms of diagnosis and therapy [38,39]. As highlighted above, recurrent skin AE symptoms are a frequent reason for referrals to dermatology or allergy consultations. The main reason behind AAE is concomitant pharmacological treatment, which should be considered the first and usually most obvious etiological factor in these cases. Medications to be considered as possible culprits in eliciting drug-induced AAE include ACE inhibitors, angiotensin receptor blockers (ARBs), gliptins (DPP-IV inhibitors), neprilysin inhibitors, direct renin inhibitors, recombinant tissue plasminogen activators, statins, non-steroidal anti-inflammatory drugs (NSAIDs), and others [40,41,42,43]. Importantly, all subjects presenting with AE in whom a hereditary background is less probable (e.g., late-onset symptoms, lack of family history) should be evaluated for other possible causes of AAE: lymphoproliferation, myeloproliferation, solid-organ tumors, and autoimmune conditions [38,39,44,45]. In general, the differential diagnosis of various forms of AE is challenging, and awareness should be raised among medical practitioners concerning the wide spectrum of its etiology [46,47].

## 4. Chronic Spontaneous and Inducible Urticaria: Pathomechanism, Classification, and Clinical Features

Chronic urticaria (CU) is a common condition with a lifetime prevalence estimated at approximately 4.4% [48,49], characterized by the development of wheals (hives), AE, or both [50]. Urticaria is predominantly a mast cell-driven condition, and mast cell-derived mediators, such as histamine, platelet-activating factor (PAF), and various cytokines, are involved in its pathogenesis [50,51]. CU can be divided into spontaneous (CSU—chronic spontaneous urticaria) and inducible (CIndU—chronic inducible urticaria) depending on whether a specific trigger for symptoms’ elicitation can be identified [52,53]. Although managed frequently by allergists, CU is not considered to be a purely allergic disease; however, an IgE-dependent sensitization profile has been ascertained in affected subjects [54,55]. Within the CIndUs, a subgroup of urticarias elicited by physical triggers has been identified. Within all CIndUs, several subtypes have been described based on the identifiable triggering factor and circumstances of occurrence, as outlined in Table 2.

AE may be an accompanying clinical feature in 40–50% of subjects affected by CU [24]. In such a case, the diagnostic and therapeutic approach is similar to that applicable in urticaria without concomitant AE. A mast cell-mediated, histaminergic AE may also occur. Such a form of AE has features indicative of mast cell involvement, i.e., timing of appearance, concomitant symptoms, family and personal history of atopy, and response to antihistamine treatment, as shown in Table 2.

## 5. Angioedema, Urticaria, and Anaphylaxis in the Context of Exercise

The majority of stimuli listed in Table 2 can be encountered in circumstances associated with exercising various sports disciplines, both indoor and outdoor, at different levels of performance. Therefore, signs and symptoms of CIndU with or without concomitant AE may occur in competitive and recreational athletes. Of note, ambient and environmental conditions are frequently associated with the presence of triggers of AE and urticaria, such as extreme temperatures, water exposure, UV light, sweating, emotional stimulation, mechanical stimulation (hits during fight sports, vibratory and mechanical stimuli due to running, and biking on uneven surfaces).

In addition, exercise-induced anaphylaxis (EIAn) creates a potential threat to subjects exercising regularly, in particular those with concomitant food allergy [19,57,58]. Skin pruritus and wheals may be the first symptoms of developing anaphylaxis, and throat and larynx edema in the course of anaphylaxis would clinically resemble symptoms of AE of other etiologies [59]. Exercise can also be a sole trigger for anaphylaxis, although data regarding such situations are not abundant [60,61,62]. More frequently, exercise is a cofactor or one of the cofactors for anaphylaxis elicited by food- or drug-related triggers [60]. The exact pathogenesis of EIAn has not been elucidated and several theories are matters of debate [63], yet food-associated triggers should always be the main suspects, particularly in relation to novel or rarely ingested foods and seemingly unexplained cases [61,64,65,66,67,68].

Co-occurrence of AE and urticaria is rather indicative of their histamine-mediated pathomechanism, although the two types of lesions do not necessarily appear at the same time. Several CIndU subtypes associate more frequently with AE symptoms. This applies mainly to cholinergic urticaria (CholU) [69], cold urticaria (ColdU) [70,71,72], and aquagenic urticaria (AquaU) [73].

## 6. Cholinergic Urticaria (CholU)

CholU symptoms commonly occur after physical activity, passive warming (e.g., hot bath, sauna), eating spicy and hot food, or emotional stress. Wheals in CholU are usually pinpoint-sized, with significant flare, and are typically localized on the skin of the upper trunk and extremities. CholU predominantly affects young males, although other subject groups may be affected as well [56].

The etiology of CholU is not fully elucidated, yet there are hypotheses suggesting an allergic reaction to own sweat, and—although not always effective—treatment with non-sedative antihistaminic agents is recommended.

In a study including 200 patients diagnosed with CholU, analysis was performed after stratification into two groups based on the age of CholU symptom onset (early onset before age 36 and late onset at the age of 36 years or older) [74]. The results of that study showed that in the early-onset group, the duration of the disease was longer compared to the late-onset group. Additionally, more allergic comorbidities, such as atopic dermatitis, were noted in the early-onset group. A type I allergic reaction to sweat with increased IgE level was associated with the early-onset group, while an autoimmune mechanism was suggested by the authors as more likely involved in the symptoms’ appearance in the late-onset group. Autoantibodies considered a possible culprit in the latter group might be directed against sweat glands, leading to pore occlusion. Autoimmune mechanism of late-onset CholU can be considered even more likely, since the majority of late-onset subjects in this study were females. Female sex has been associated with being more prone to develop autoimmune reactions, including the ones linked to CSU, e.g., autoimmune thyroid disease, and this feature tends to increase with age [75,76]. Concerning AE, there were no differences observed between early- and late-onset CholU subjects. The frequency of occurrence of at least one AE attack was 24.5% out of 200 study subjects.

The exact incidence of edema symptoms in the course of CholU has not been established in systematic prospective studies. Mellerowicz and collaborators [69] retrospectively analyzed clinical data from 107 CholU patients followed at the UCARE (Urticaria Network of Reference and Excellence) Center in Berlin. At least one occurrence of symptoms of AE associated with CholU was observed in 45.8% of patients. No predisposition related to gender or other demographic factors was observed. The occurrence of AE symptoms associated with CholU was not dependent on the total serum IgE concentration either. AE was more common (but not statistically significantly) in patients with concomitant allergy and atopic dermatitis. However, no association of AE symptoms with the diagnosis of allergic rhinitis or contact dermatitis was observed.

In that group of patients, the occurrence of AE symptoms in the course of cholinergic urticaria was also associated with a higher frequency of extracutaneous post-exercise symptoms, such as dizziness, a feeling of dyspnea and general discomfort, and tachycardia.

## 7. Cold Urticaria (ColdU) and Cold-Induced Anaphylaxis (ColdA)

ColdU is characterized by the rapid-onset appearance of wheals, AE, or both in response to various cold triggers, including exposure to cold air, contact with fluids or solid surfaces, and ingestion of cold foods or drinks [56]. Therefore, this type of inducible urticaria is potentially associated with a wide spectrum of sports activities associated either with exposure to cold water or with exercising in outdoor winter sport disciplines. Its mechanisms, as those of other CIndUs, remain not fully elucidated; however, ColdU should focus the attention of all medical personnel working with athletes due to the considerable risk of AE and anaphylaxis it conveys.

The annual incidence of ColdU in Europe is estimated at 0.05%. Estimations of the incidence of cold-induced anaphylaxis occurring (ColdA) in ColdU subjects are limited due to a lack of a uniform definition. Anaphylaxis was diagnosed in 37% of ColdU subjects in a COLD-CE study [77]; however, the reported frequency of systemic reactions of different characteristics and diagnosed based on different definitions differs among sources, having been ascertained in 4% to up to 51% of ColdU patients (reviewed in [71]).

Cold-induced AE is one of the risk factors for anaphylactic reactions in ColdU subjects [72,77]. In addition, oropharyngeal swelling, obstruction, or discomfort may appear after drinking cold water [78]. These symptoms may, however, be purely subjective and result from the irritation of mucosae by cold stimuli and should be interpreted with caution. Gastrointestinal symptoms appearing after cold drink ingestion may equally be sequelae of cooling and irritation of mucosae and not the hallmark of developing abdominal AE [77]. Predisposition to anaphylaxis in ColdU has been associated with several clinical and laboratory features. Hymenoptera venom-induced anaphylaxis, elevated total serum IgE, hereditary α-tryptasemia (HαT), and the presence of the *KIT* gene D816V mutation characteristic of mastocytosis have been associated with increased risk of ColdA [72]. Increased risk of anaphylaxis associated with ColdU underlines the necessity of epinephrine prescription for all affected patients and the need for awareness raising and educational activities targeting the general public and medical professionals [79,80].

## 8. Aquagenic Urticaria (AquaU) and Angioedema

This type of inducible urticaria is characterized by the appearance of itchy wheals after contact with water during, for instance, swimming or bathing. Wheals appear within 30 min after exposure and tend to last for 30–60 min, and are usually localized at the trunk, despite other body areas having been equally exposed to water. This feature seems characteristic of AquaU and possibly distinct from other CIndUs, which may help direct the diagnostic pathway. Wheals in AquaU are morphologically similar to the ones observed in CholU. The occurrence of AE has been described in AquaU patients, while other possible concomitant features include dizziness, breathing difficulty, headache, and anaphylaxis. In addition, AquaU patients should be evaluated for polycythemia vera, myelodysplastic syndromes, viral infections (hepatitis C, HIV), non-Hodgkin lymphoma, and hypereosinophilic syndrome, since these conditions may initially present as AquaU [73,81,82,83].

Rujitharanawong et al. [73] performed a systematic review of published aquagenic urticaria and aquagenic AE case reports and case series presenting 77 patients. Based on clinical data from published cases, they proposed dividing AquaU into two subtypes: familial (FAquaU) and acquired (AAquaU). In FAquaU, patients had a positive family history of AquaU and were usually younger than patients with AAquaU at symptoms’ onset.

As revealed by this systematic review, AE symptoms in AquaU are quite frequent. They have not been reported in familial AquaU, whereas among 63 identified acquired AquaU subjects, one had isolated AE without wheals, and in four AE was an accompanying feature. Notably, in two patients, AE was caused by water ingestion and not external exposure. Isolated AE associated solely with water exposure, ascertained in a single patient during sports activity, was accompanied by pruritus, but without concomitant urticarial wheals. Notably, provocation tests did not elicit symptoms typical of cholinergic urticaria, which further confirmed the diagnosis of such a rare form of AE. In that case, AE induced by water exposure tended to subside over several subsequent weeks, enabling the patient to resume sporting activities [84].

## 9. Vibratory Angioedema

Vibratory angioedema (VA)—also classified as a CIndU—is caused by mechanical stimuli to which the body is exposed during exercise. The most common circumstances in which VA symptoms occur include:

- Running.
- Riding a bike, motorcycle, driving a car (especially on uneven terrain).
- Massage.
- Contact with water jets in the shower.
- Vigorous rubbing of the body with a towel.

Individual case reports of vibratory edema include skiing, bowling, undergoing dental procedures, as well as snoring and sexual intercourse as stimuli leading to its occurrence [85].

Congenital and acquired (sporadic) forms of VA have been described [85,86,87]. The congenital form of VA is of autosomal-dominant inheritance and has been described in 28 members of four families [87,88,89]. The presence of congenital VA is associated with the presence of mutations in the *ADGRE2* gene, which encodes a protein involved in the regulation of the release of mediators of allergic reactions, for example, from mast cells.

The most complete review of VA cases published in the medical literature and a proposed classification was presented by Kulthanan and colleagues [85]. Based on a systematic review of 83 case reports, these authors estimated that about a third of VA cases are congenital. The occurrence of isolated edema symptoms without accompanying urticaria is observed more often in the acquired form than in congenital VA (39.5% vs. 14%). Other characteristics of the acquired (non-hereditary) form of vibratory edema are:

- Onset in adulthood.
- Less frequent occurrence of systemic (extracutaneous) symptoms.
- The most common stimuli causing symptoms: cycling, motorcycle, driving a car.
- Less frequent occurrence during running, brisk walking, and massage.
- Often a positive family history of atopy.

In the congenital form of VA, the onset of symptoms was earlier and systemic reactions were more common. Triggers were also different in both groups: for example, jogging, running, fast walking, and massage were the triggering factors in patients with hereditary VA, while more simple activities such as using a towel or taking a shower caused AE only in the hereditary group. On the other hand, cycling, riding a motorcycle, and driving a car were exclusively triggering symptoms in acquired VA.

## 10. Exercise-Induced AE as a Manifestation of an IgE-Mediated Reaction

Epidemiological studies show that anaphylaxis will occur at least once in a lifetime in 0.3–5.1% of the general population depending on the definitions used, study methodology, and geographical areas, and its mortality rate is estimated at 0.05–0.51 per million people/year, varying in relation to the causing factor [18]. According to various estimates, between 2% and 15% of anaphylactic reactions may be directly caused by or be associated with performing physical exercise. As mentioned above, exercise is a cofactor of anaphylactic reaction, accompanying it in 10–15% of cases [90]. Exercise-induced anaphylaxis (EIAn) is a rare, unpredictable, potentially fatal syndrome in which a systemic allergic reaction occurs in combination with exercise or physical activity. Exercise-induced anaphylaxis may occur independently of food allergen ingestion or may be associated with food allergen ingestion before or after exercise, in which case it is called food-dependent exercise-induced anaphylaxis (FDEIAn).

A systematic review of the literature published in 2022 by Kulthanan et al. [91] included over 200 papers (43 cohort studies, 15 case series, 173 single-case reports) describing symptoms of urticaria, AE, and anaphylaxis induced by exercise and associated with food allergy. It is worth noting that the papers included in the review concerned cases confirmed by a provocation test. The analyzed data came from 722 patients whose median age was 25 years, and of whom 55% were male.

Symptoms of exercise-induced anaphylaxis associated with food allergy (FDEIAn) occurred in almost 80% of reported cases. The characteristics and frequency of urticaria and edema symptoms in the course of FDEIAn were as follows:

- Urticaria without AE—55.1%.
- Urticaria and AE—37.7%.
- AE without urticaria—7.1%.

Exercise-induced urticaria–edema lesions without signs of anaphylaxis occurred in 16.6% of the cases included in this review. Individual configurations of clinical symptoms occurred with the following frequency:

- Urticaria without AE—56.7%.
- Urticaria and AE—40.8%.
- AE without urticaria—2.5%.

Urticaria is not only a comorbidity symptom in the vast majority of cases of exercise-induced AE but is also a factor present in the medical history in more than a third of patients experiencing post-exercise anaphylaxis, urticaria, and AE.

Isolated symptoms of post-exercise AE without concomitant urticaria eruptions occur based on data from Kulthanan and colleagues [91], usually as one of the components of an exercise-induced anaphylactic reaction, but in individual cases, they are the only symptom of a post-exercise allergic reaction. The rare occurrence of isolated post-exercise AE is also confirmed by data from studies by other authors.

Symptoms of exercise-induced AE may also coexist with symptoms of respiratory discomfort typical of asthma. Describing the case of a 15-year-old boy who had recurrent swelling in the face and around the eyes accompanied by respiratory discomfort in direct temporal relationship with intense physical exercise (e.g., after several dozen minutes of playing baseball), Leung and Hegde [92] concluded that exercise-induced comorbidity of asthma and AE should be distinguished from those of exercise-induced anaphylaxis.

An extensive case report by Magen and Chikovani [93] describes a young woman suffering from AE without urticaria appearing during or shortly after exercise, but only in relation to consumption of milk up to a few hours before. A skin-prick test (SPT) with lactalbumin, casein, and cow’s milk was positive, and specific IgE to cow’s milk was elevated. Several provocations were performed, but only exercise following milk consumption led to AE, which responded to fexofenadine. The postulated mechanisms of its symptoms include:

- Lowering the activation threshold of mast cells and basophils leads to their degranulation and release of histamine and vasoactive mediators.
- Changes in the permeability of the gastrointestinal epithelium resulting in easier uptake of food allergens.
- Dysregulation of the autonomic nervous system.
- Increased blood supply to the muscles and thus greater exposure of mast cells in muscle tissue to food allergens.

The authors of that case report suggest that many cases of histaminergic AE (without accompanying urticaria) classified as “idiopathic” are in fact exercise-related. It can be difficult to establish a causal relationship between exercise and the onset of AE, especially if the onset of swelling was delayed and symptoms were not significantly severe. All the more reason to emphasize the need to carefully collect medical history regarding the circumstances of the reaction.

Caffarrelli et al. [94] reported a case of an 8-year-old boy who experienced three episodes of facial AE, and tongue tingling about 15 min after football training sessions, and in all cases approximately 5 min after eating a tomato pizza containing tomato, milk, and wheat as suspected culprit allergen sources. Symptoms resolved after antihistamine treatment. The medical history of the patient indicated allergic rhinitis (AR) and early childhood asthma. Skin-prick tests were positive for several allergens: soy, corn, peas, tomato, carrot, grass, dust, hazel, mugwort, and plantain. Serum sIgE to grass pollen and dust mite allergens were detected. In an attempt to find a trigger, several exercise challenge tests were performed under different conditions: after 12 h of fasting; after eating cow’s milk and bread; after eating fresh tomatoes; and 5 min before eating fresh tomatoes. The challenge consisted of 6 min of indoor free running, increasing the heart rate to at least 170 beats per minute. Symptoms (facial AE and conjunctivitis) appeared only after fresh tomato consumption at the end of exercise. No breathing difficulty or drop in blood pressure was observed. In this case, the causal relationship between exercise-induced AE and food allergy was convincingly confirmed through a provocation test in controlled conditions.

Interesting reports on two cases of post-exercise AE caused by thermal stimuli (thermal angioedema) have been provided by Japanese authors [95,96]. Both accounts referred to females approximately 20 years of age with polyvalent IgE-mediated allergy and atopic dermatitis. Symptoms of isolated (i.e., without concomitant urticaria) asymmetric eyelid edema accompanied by gradually increasing respiratory discomfort occurred with an increase in body temperature during exercise or bathing. This relationship was confirmed through provocation testing. The authors of these case reports speculate that this form of AE may be an incomplete form of cholinergic urticaria, which may be caused by—among others—an increase in body temperature during physical exertion.

## 11. HAE Affects Training and Sports Performance

Trauma and physical stimuli belong to potential triggers of HAE attacks; therefore, sports activity is one of the everyday life domains considerably influenced by HAE symptoms. Given the rarity of the disease, reports and studies related to the effect of HAE on sports activity and career are not numerous.

Zarnowski and Treudler [97] surveyed 30 adult HAE patients (77% females) regarding dietary and physical triggers related to HAE symptoms. In sum, 21 out of 30 subjects declared regular recreational physical activity, mainly cycling, running, hiking, and strength training. Worsening of HAE symptoms due to recreational exercise was reported by 62% of physically active survey participants. Six of them experienced HAE attacks due to mechanical pressure on palmoplantar areas during hiking or strength training, two reported genital swelling after intensive cycling, and one reported an abdominal attack due to emotional stress and fear when climbing.

In a long-term Hungarian study [98] where the observation period of adolescent and adult HAE patients spanned more than 7 years, physical exertion was identified as one of the crucial triggers for edema. Exercise was mostly associated with the occurrence of subcutaneous attacks, with single cases of abdominal attacks and no upper-airway edema reported in association with physical exertion.

Similar findings were reported in a Swedish retrospective study [99] involving 36 children aged 1–17 years. The most frequent manifestation of HAE attacks triggered by sports activity and trauma was skin swelling, while in a single case, those stimuli were identified as triggers of abdominal HAE symptoms.

Ashrafian [100] described a family where in three generations, males with symptoms highly suggestive of HAE practiced martial arts (one of kung fu style) and psychological techniques aiming to decrease aggressiveness, reduce the symptoms of stress, and increase mood, known as qigong. All concerned family members suffered from attacks of acute abdominal pain and acute laryngeal edema. Two members experienced near-fatal asphyxia immediately following sports activity, and another two were subject to appendectomy due to abdominal pain, which was not related to exercise. One of the third-generation members died at age 26 of acute laryngeal edema classified as anaphylaxis. However, symptoms were unresponsive to steroids and epinephrine, and no culprit allergen was identified. HAE diagnosis was confirmed through laboratory testing only in a fourth-generation male member of that family, who is, however, not pursuing the family tradition and does not practice on a competitive basis. He was put on pharmacoprophylaxis, and recreational football playing did not elicit HAE attacks.

## 12. Treatment and Management of Exercise-Induced AE

The effectiveness of treatment of the symptoms of exercise-induced AE is determined by the pathogenesis of symptoms and the circumstances in which they tend to occur. Additionally, the hereditary aspect of angioedema in an exercising subject should also be considered upon evaluation, and if applicable, relevant prophylaxis and treatment measures should be implemented. It should be specifically underlined that standard treatment of histamine-induced reactions (i.e., H1 histamine receptor antagonists and glucocorticosteroids), which are beneficial in exercise-induced angioedema associated with mast cell mediator release with or without concomitant IgE-mediated allergy, are not effective in HAE, which is one of the characteristic features differentiating this form of AE from others discussed above. Additionally, although the “hereditary” feature of HAE implies a positive family history, one should bear in mind that approximately 15–20% of confirmed HAE cases are diagnosed de novo, without precedent cases reported in the family. Symptoms of HAE with normal C1-INH, i.e., those with mutations in genes other than *SERPING1* (see above—Section 3) are posing considerable diagnostic challenges and should be considered possible differential diagnosis in otherwise unexplained cases of AE.

The basic and universal recommendation is to avoid exercise as well as additional factors and circumstances that cause symptoms. Optimal application of such a recommendation requires the identification of the factor causing the swelling and the cofactors contributing to the onset of symptoms. Due to the variability in symptoms of post-exercise AE and often impossible identification of triggers, it is also necessary to provide patients with post-exercise AE with a prescription for self-administered epinephrine.

Management of patients with symptoms of histaminergic post-exercise AE associated with IgE-mediated allergy includes the use of second-generation antihistamines. Post-exercise AE induced by a change in temperature (thermal angioedema) is characterized, similarly to CholU, by a poor response to antihistamines. Similarly, the limited effectiveness of both first- and second-generation antihistamines is observed in the case of VA.

The following drugs have also been used to treat various forms of exercise-related AE:

- Systemic glucocorticoids.
- Antileukotriene drugs.
- Disodium cromoglycate.
- Immunosuppressants (cyclosporine A, methotrexate).
- Anti-IgE monoclonal antibody (omalizumab).

Currently, there are no clinical trial results for the treatment or prevention of exercise-related AE that meet the criteria of evidence-based medicine (EBM). Information on the effectiveness of individual drugs in patients with symptoms of this type of edema usually comes from case reports or case series summaries, as outlined above. The picture of possible approach options to exercise-induced AE may be further blurred and complicated by the fact that a significant number of the aforementioned reports describe cases of post-exercise swelling with concomitant urticarial symptoms.

In cases of confirmed diagnosis of HAE, relevant on-demand and prophylactic modalities should be provided, as described in previously published guidelines [3]. Currently recommended on-demand and prophylactic management options are presented in Table 3. One should also bear in mind that recent years have witnessed substantial progress in the development of treatment and prophylaxis of HAE, and updated recommendations should be applied [101,102,103]. Apart from the recently approved garadacimab [104,105,106] (already included in Table 3), several new drugs are in the pipeline or awaiting approval over next few months. Sebetralstat is expected to shortly become the next licensed option for on-demand HAE management, being the first modern drug to be taken orally for treatment of acute HAE attacks [107,108]. However, the accessibility of recommended tools may differ depending on the economic setting [109,110,111], which should be accounted for while implementing the guidelines for HAE treatment and prophylaxis.

## 13. Summary

Urticarial symptoms have been reported quite frequently and consistently associated with various exercise stimuli. Usually, those symptoms are accompanied by AE; however, when the affected subjects present with only AE as an isolated sign, the diagnostic workup becomes more challenging for the physicians. This phenomenon may be induced by various mechanisms, and thus requires a more detailed assessment. Late-onset isolated AE always suggests the existence of concomitant lymphoproliferation, myelodysplasia, autoimmune pathology, and possibly a drug-related etiology. HAE is a rare condition that usually exacerbates upon exercising or due to mechanical trauma. Therefore, awareness of the possibility of the occurrence of angioedema symptoms provoked by various triggers should be raised both among sport medicine professionals and the athletes themselves.

## Figures and Tables

**Table 2:** Subtypes of chronic inducible urticaria (CIndU) and their selected triggers (based on [24,50,53,56], modified).

CIndU Subtype	Relevant Trigger(s)
*Physical urticaria*	
Symptomatic dermographism	Mechanical stroking, scratching, or rubbing and similar stimuli
Cold urticaria (ColdU)	Exposure to cold environment, contact with cold objects, liquids, surfaces, cooling/rewarming
Delayed pressure urticaria (DPU)	Vertical pressure applied upon given area of the skin, e.g., straps, belts, backpacks, etc.
Solar urticaria	Exposure to sunlight of specific wavelengths
Heat urticaria	Local skin exposure to heating
Vibratory angioedema	Vibration of different origin (mechanical tools, exercise on uneven surface—among others, running, mountain biking)
*Other inducible urticaria*	
Cholinergic urticaria (CholU)	Sweating, increase in body temperature sequel to exercise, warm bath, sauna, ingestion of hot and spicy foods, emotional stress
Aquagenic urticaria	Exposure to water at any temperature
Contact urticaria	Direct contact with urticariogenic factor, such as chemical element or compound

**Table 3:** Summary of currently recommended options for on-demand treatment of HAE attacks and for short-term and long-term prophylaxis. Prepared based on information provided in most recent WAO/EAACI guidelines [3]. Information related to garadacimab (approved in 2025 and not mentioned in the guideline) based on relevant publications. Abbreviations (if not explained in the table): HBV, hepatitis B virus; HCV, hepatitis C virus; HIV, human immunodeficiency virus; QD, daily; Q2W, every two weeks; Q4W, every four weeks; Q1M, once a month; DVT, deep vein thrombosis.

Therapeutic Agent	Route of Administration	Frequency of Administration (for Prophylactic Agents)	Mechanism of Action	Remarks
*On-demand treatment*				
Plasma-derived C1 inhibitor (pdC1-inh)	Intravenous	n.a.	Substitution of deficient C1 inhibitor	Good safety and tolerability profile, negligible risk of adverse reactions; no HBV, HCV or HIV transmission reported.
Recombinant C1-inhibitor (rhC1-inh)	Intravenous	n.a.	Substitution of deficient C1 inhibitor	Recommended in adults and children aged ≥ 2 years. Not recommended in pregnancy and lactation.
Icatibant	Subcutaneous	n.a.	Bradykinin B2 receptor antagonist	Recommended in adults and children aged ≥ 2 years. To be administered with caution during pregnancy. Refraining from breastfeeding recommended within 12 h after injection.
Ecallantide	Subcutaneous	n.a.	Kallikrein inhibitor	Safety concerns: considerable risk of serious hypersensitivity reactions, anaphylaxis reported in 3–4% of treated subjects. Licensed in the US and some Latin American countries. Recommended in adults and adolescents aged ≥ 12 years.
Fresh frozen plasma (FFP); solvent detergent-treated plasma (SDP)	Intravenous	n.a.	Substitution of deficient C1 inhibitor	To be administered only if pd-C1-inh, rC1-inh, icatibant. or ecallantide not available. Risk of transmission blood-borne disease and allosensitization to be taken into consideration.
*Short-term prophylaxis*				
Plasma-derived C1 inhibitor (pd-C1inh)	Intravenous	Recommended to be administered before medical interventions involving mechanical impact on airways or gastrointestinal tract mucosae, and before surgical and dental interventions—as close to the procedure as possible	Substitution of deficient C1 inhibitor	Dosage depending on product approval, may vary between countries.
Recombinant C1 inhibitor (rh-C1inh)	Intravenous	Indications as for pd-C1inh	Substitution of deficient C1 inhibitor	Less evidence confirming prophylactic effect, may be considered if pd-C1inh not available.
Fresh frozen plasma (FFP)	Intravenous	Indications as for pd-C1inh	Substitution of deficient C1-inhibitor	Second-line prophylaxis if pd-C1inh not available, safety concerns regarding blood-borne disease transmission and allosensitization
Attenuated androgens (danazol, stanozolol)	Oral	5 days before and 2–3 days post-procedure	Increase of C4 and C1-inhibitor synthesis; promotion of bradykinin degradation	Risk of side-effects associated with long-term use (see below), if repeated courses prescribed. To be used only if C1 inhibitor not accessible.
*Long-term prophylaxis*				
Plasma-derived C1 inhibitor (pdC1-inh)	Subcutaneous	Twice a week	Substitution of deficient C1 inhibitor	Recommended as one of the options for first-line long-term prophylaxis. Interval between doses may be modified if good symptom control achieved, as assessed through patient-reported outcome measures (PROMs).
Lanadelumab	Subcutaneous	Q2W or Q4W	Monoclonal antibody against plasma kallikrein	Recommended as one of the options for first-line long-term prophylaxis. Interval between doses may be modified if good symptom control achieved, as assessed through patient-reported outcome measures (PROMs).
Berotralstat	Oral	QD	Inhibitor of plasma kallikrein proteolytic activity	Recommended as one of the options for first-line long-term prophylaxis; gastrointestinal side effects may incur dose reduction. Preferred by patients due to oral route of administration.
Garadacimab	Subcutaneous	Q1M	Monoclonal antibody against activated factor XII (FXII)	Approval received January–February 2025 in the UK, Australia, Japan, Switzerland, and the European Union. FDA approval pending.
Attenuated androgens	Oral	QD	Increase of C4 and C1-inhibitor synthesis; promotion of bradykinin degradation	Long-term use associated with considerable side effects: virilization and menstrual disturbances (in females), diminished libido, weight gain, headache, myalgia, depression, and acne. Absolutely contraindicated in pregnancy due to risk of virilization of female fetus.
Antifibrinolytics (e.g., tranexamic acid)	Oral	QD		To be used only if first-line treatment not available and androgens are contraindicated. Good safety profile, but to be used with caution in patients with thrombophilia or increased risk of thrombotic events, such as DVT or pulmonary embolism.

## Data Availability

No new data were created during preparation of this review.

## Abbreviations

The following abbreviations are used in this manuscript:

AE	angioedema
BK	bradykinin
C1INH	C1-esterase inhibitor
CholU	cholinergic urticaria
CIndU	chronic inducible urticaria
FDEIAn	food-dependent exercise-induced anaphylaxis
HAE	hereditary angioedema
VA	vibratory angioedema

## References

[1] Reshef A., Buttgereit T., Betschel S.D., Caballero T., Farkas H., Grumach A.S., Hide M., Jindal A.K., Longhurst H., Peter J. (2024). Definition, Acronyms, Nomenclature, and Classification of Angioedema (DANCE): AAAAI, ACAAI, ACARE, and APAAACI DANCE Consensus. J. Allergy Clin. Immunol..

[2] Cicardi M., Aberer W., Banerji A., Bas M., Bernstein J.A., Bork K., Caballero T., Farkas H., Grumach A., Kaplan A.P. (2014). Classification, Diagnosis, and Approach to Treatment for Angioedema: Consensus Report from the H Ereditary A Ngioedema I Nternational W Orking G Roup. Allergy.

[3] Maurer M., Magerl M., Betschel S., Aberer W., Ansotegui I.J., Aygören-Pürsün E., Banerji A., Bara N., Boccon-Gibod I., Bork K. (2022). The International WAO/EAACI Guideline for the Management of Hereditary Angioedema—The 2021 Revision and Update. Allergy.

[4] Kaplan A.P. (2008). Angioedema. World Allergy Organ. J..

[5] Jalaj S., Scolapio J.S. (2013). Gastrointestinal Manifestations, Diagnosis, and Management of Hereditary Angioedema. J. Clin. Gastroenterol..

[6] Patel N., Suarez L.D., Kapur S., Bielory L. (2015). Hereditary Angioedema and Gastrointestinal Complications: An Extensive Review of the Literature. Case Rep. Immunol..

[7] Staller K., Lembo A., Banerji A., Bernstein J.A., Shah E.D., Riedl M.A. (2022). Consider Hereditary Angioedema in the Differential Diagnosis for Unexplained Recurring Abdominal Pain. J. Clin. Gastroenterol..

[8] Rubinstein E., Stolz L.E., Sheffer A.L., Stevens C., Bousvaros A. (2014). Abdominal Attacks and Treatment in Hereditary Angioedema with C1-Inhibitor Deficiency. BMC Gastroenterol..

[9] Longhurst H.J., Bork K. (2019). Hereditary Angioedema: An Update on Causes, Manifestations and Treatment. Br. J. Hosp. Med..

[10] Nzeako U.C., Longhurst H.J. (2012). Many Faces of Angioedema: Focus on the Diagnosis and Management of Abdominal Manifestations of Hereditary Angioedema. Eur. J. Gastroenterol. Hepatol..

[11] Stobiecki M., Obtulowicz P., Porebski G., Dyga W., Czarnobilska E., Obtulowicz K. (2019). Severe Abdominal HAE Attacks: An Analysis of 7 Cases. Arch. Clin. Med. Case Rep..

[12] Obtulowicz P., Stobiecki M., Dyga W., Popiela T., Obtulowicz K. (2024). Abdominal Attack in a Patient with Hereditary Angioedema Due to C1 Inhibitor Deficiency Complicated by a Perforated Peptic Ulcer. Pol. J. Allergol..

[13] Banerji A., Li Y., Busse P., Riedl M.A., Holtzman N.S., Li H.H., Davis-Lorton M., Bernstein J.A., Frank M., Castaldo A.J. (2018). Hereditary Angioedema from the Patient’s Perspective: A Follow-up Patient Survey. Allergy Asthma Proc..

[14] Papadopoulos N.G., Agache I., Bavbek S., Bilo B.M., Braido F., Cardona V., Custovic A., de Monchy J., Demoly P., Eigenmann P. (2012). Research Needs in Allergy: An EAACI Position Paper, in Collaboration with EFA. Clin. Transl. Allergy.

[15] Seys S.F., Quirce S., Agache I., Akdis C.A., Alvaro-Lozano M., Antolín-Amérigo D., Bjermer L., Bobolea I., Bonini M., Bossios A. (2019). Severe Asthma: Entering an Era of New Concepts and Emerging Therapies: Highlights of the 4th International Severe Asthma Forum, Madrid, 2018. Allergy.

[16] Agache I., Akdis C., Akdis M., Al-Hemoud A., Annesi-Maesano I., Balmes J., Cecchi L., Damialis A., Haahtela T., Haber A.L. (2024). Climate Change and Allergic Diseases: A Scoping Review. J. Clim. Change Health.

[17] Suzuki K. (2019). Chronic Inflammation as an Immunological Abnormality and Effectiveness of Exercise. Biomolecules.

[18] Cardona V., Ansotegui I.J., Ebisawa M., El-Gamal Y., Fernandez Rivas M., Fineman S., Geller M., Gonzalez-Estrada A., Greenberger P.A., Sanchez Borges M. (2020). World Allergy Organization Anaphylaxis Guidance 2020. World Allergy Organiz. J..

[19] Carlisle A., Lieberman J.A. (2024). Getting in Shape: Updates in Exercise Anaphylaxis. Curr. Allergy Asthma Rep..

[20] DuToit G., Smith P., Muraro A., Fox A.T., Roberts G., Ring J., Worm M. (2024). Identifying Patients at Risk of Anaphylaxis. World Allergy Organ. J..

[21] Farkas H., Balla Z., Riedl M.A. (2022). Differentiating Histaminergic and Nonhistaminergic Angioedema with or without Urticaria. J. Allergy Clin. Immunol..

[22] Ferrer M., Rodriguez-Garijo N., Sabaté-Brescó M. (2023). Medical Algorithm: Diagnosis and Management of Histaminergic Angioedema. Allergy.

[23] Sabaté-Brescó M., Rodriguez-Garijo N., Azofra J., Baeza M.L., Donado C.D., Gaig P., Guilarte M., Herrera-Lasso V., Labrador-Horrillo M., Sala-Cunill A. (2021). A Comparative Study of Sex Distribution, Autoimmunity, Blood, and Inflammatory Parameters in Chronic Spontaneous Urticaria with Angioedema and Chronic Histaminergic Angioedema. J. Allergy Clin. Immunol. Pract..

[24] Busse P.J., Smith T. (2017). Histaminergic Angioedema. Immunol. Allergy Clin. N. Am..

[25] Donaldson V.H., Evans R.R. (1963). A Biochemical Abnormality in Hereditary Angioneurotic Edema. Am. J. Med..

[26] Maurer M., Magerl M. (2021). Differences and Similarities in the Mechanisms and Clinical Expression of Bradykinin-Mediated vs. Mast Cell–Mediated Angioedema. Clin. Rev. Allergy Immunol..

[27] Drouet C., López-Lera A., Ghannam A., López-Trascasa M., Cichon S., Ponard D., Parsopoulou F., Grombirikova H., Freiberger T., Rijavec M. (2022). SERPING1 Variants and C1-INH Biological Function: A Close Relationship with C1-INH-HAE. Front. Allergy.

[28] Parsopoulou F., Loules G., Zamanakou M., Csuka D., Szilagyi A., Kompoti M., Porebski G., Psarros F., Magerl M., Valerieva A. (2022). Searching for Genetic Biomarkers for Hereditary Angioedema Due to C1-Inhibitor Deficiency (C1-INH-HAE). Front. Allergy.

[29] Farkas H., Germenis A.E., Longhurst H. (2022). Editorial: C1 Inhibitor Deficiency and Angioedema. Front. Allergy.

[30] Zuraw B.L., Bork K., Bouillet L., Christiansen S.C., Farkas H., Germenis A.E., Grumach A.S., Kaplan A., López-Lera A., Magerl M. (2025). Hereditary Angioedema with Normal C1 Inhibitor: An Updated International Consensus Paper on Diagnosis, Pathophysiology, and Treatment. Clin. Rev. Allergy Immunol..

[31] Adatia A., Boursiquot J.-N., Goodyear D., Kalicinsky C., Kanani A., Waserman S., Nguyen M.M.L., Wadhwa A., Weiss J., El-Zoeiby A. (2024). Real-World Outcomes of Patients with Hereditary Angioedema with Normal C1-Inhibitor Function and Patients with Idiopathic Angioedema of Unknown Etiology in Canada. Allergy Asthma Clin. Immunol..

[32] Loules G., Parsopoulou F., Zamanakou M., Csuka D., Bova M., González-Quevedo T., Psarros F., Porebski G., Speletas M., Firinu D. (2020). Deciphering the Genetics of Primary Angioedema with Normal Levels of C1 Inhibitor. J. Clin. Med..

[33] Rozevska M., Kanepa A., Purina S., Gailite L., Nartisa I., Farkas H., Rots D., Kurjane N. (2024). Hereditary or Acquired? Comprehensive Genetic Testing Assists in Stratifying Angioedema Patients. Allergy Asthma Clin. Immunol..

[34] Santacroce R., D’Andrea G., Maffione A.B., Margaglione M., d’Apolito M. (2021). The Genetics of Hereditary Angioedema: A Review. J. Clin. Med..

[35] D’Apolito M., Santacroce R., Vazquez D.O., Cordisco G., Fantini C.A., D’Andrea G., Leccese A., Colia A.L., Martinez P., Zanichelli A. (2024). DAB2IP Associates with Hereditary Angioedema: Insights into the Role of VEGF Signaling in HAE Pathophysiology. J. Allergy Clin. Immunol..

[36] Rupar N., Šelb J., Košnik M., Zidarn M., Andrejević S., Čulav L., Grivčeva-Panovska V., Korošec P., Rijavec M. (2024). The CC2D2B Is a Novel Genetic Modifier of the Clinical Phenotype in Patients with Hereditary Angioedema Due to C1 Inhibitor Deficiency. Gene.

[37] Giavina-Bianchi P., Giavina-Bianchi M., Kalil J. (2025). Urticaria Unveiled in Hereditary Angioedema with Carboxypeptidase N Mutation. J. Allergy Clin. Immunol. Glob..

[38] Valle S.O.R., Alonso M.L.O., Dortas Junior S.D., Goudouris E.S., De Carvalho A.L.R.B., Capelo A.V., Mansour E., Bernardes A.F., Leite L.F.B., Giavina-Bianchi P. (2022). Acquired Angioedema Due to C1-Inhibitor Deficiency: A Challenging Condition. Int. Arch. Allergy Immunol..

[39] Pólai Z., Balla Z., Andrási N., Kőhalmi K.V., Temesszentandrási G., Benedek S., Varga L., Farkas H. (2021). A Follow-up Survey of Patients with Acquired Angioedema Due to C1-inhibitor Deficiency. J. Intern. Med..

[40] Lacuesta G., Betschel S.D., Tsai E., Kim H. (2024). Angioedema. Allergy Asthma Clin. Immunol..

[41] Smolinska S., Antolín-Amérigo D., Popescu F.-D. (2023). Bradykinin Metabolism and Drug-Induced Angioedema. Int. J. Mol. Sci..

[42] Hahn J., Greve J., Bas M., Kojda G. (2023). Bradykinin-Mediated Angioedema Induced by Commonly Used Cardiovascular Drugs. Drugs Drug Candidates.

[43] Kumar Y.S., Green K., Roache R., Rodrigues S. (2024). Fluvastatin-Induced Angioedema: A Rare Case Study and Literature Review. Discov. Med..

[44] Bork K., Staubach-Renz P., Hardt J. (2019). Angioedema Due to Acquired C1-Inhibitor Deficiency: Spectrum and Treatment with C1-Inhibitor Concentrate. Orphanet J. Rare Dis..

[45] Zając M., Bożek A., Kozłowska R., Grzanka A. (2023). Acquired Angioedema in Selected Neoplastic Diseases. Medicina.

[46] Pines J.M., Poarch K., Hughes S. (2021). Recognition and Differential Diagnosis of Hereditary Angioedema in the Emergency Department. J. Emerg. Med..

[47] Evcen R., Çölkesen F., Aykan F.S., Kılınç M., Yıldız E., Ergün Ü.Y., Önalan T., Kahraman S., Gerek M.E., Arslan Ş. (2024). Awareness of Hereditary Angioedema among Emergency Physicians: A Survey Study. Pol. J. Allergol..

[48] Fricke J., Ávila G., Keller T., Weller K., Lau S., Maurer M., Zuberbier T., Keil T. (2020). Prevalence of Chronic Urticaria in Children and Adults across the Globe: Systematic Review with Meta-analysis. Allergy.

[49] Ben-Shoshan M., Kanani A., Kalicinsky C., Watson W. (2024). Urticaria. Allergy Asthma Clin. Immunol..

[50] Zuberbier T., Abdul Latiff A.H., Abuzakouk M., Aquilina S., Asero R., Baker D., Ballmer-Weber B., Bangert C., Ben-Shoshan M., Bernstein J.A. (2022). The International EAACI/GA^2^LEN/EuroGuiDerm/APAAACI Guideline for the Definition, Classification, Diagnosis, and Management of Urticaria. Allergy.

[51] Kulthanan K., Church M.K., Grekowitz E.M., Hawro T., Kiefer L.A., Munprom K., Nanchaipruek Y., Rujitharanawong C., Terhorst-Molawi D., Maurer M. (2022). Evidence for Histamine Release in Chronic Inducible Urticaria–A Systematic Review. Front. Immunol..

[52] Bizjak M., Košnik M. (2024). Key Differences between Chronic Inducible and Spontaneous Urticaria. Front. Allergy.

[53] Maurer M., Bonnekoh H., Grekowitz E., Kiefer L., Munoz M., Pereira M.P., Terhorst-Molawi D. (2024). An Algorithm for the Diagnosis and Treatment of Chronic Inducible Urticaria, 2024 Update. Allergy.

[54] Yang X., Li S., Chen A., Wang H., Deng S., Ni B., Song Z., Chen Q. (2024). Distinct IgE Sensitization Profiles in Chronic Urticaria: A Comparative Study with Classic Allergic Diseases. Front. Immunol..

[55] Zhang J., Tang Y., Yang D., Yu J. (2025). Investigating Allergen-Specific IgE Distribution and Correlations in Chronic Urticaria: A Retrospective Study in Shanghai, China. Eur. J. Med. Res..

[56] Muñoz M., Kiefer L.A., Pereira M.P., Bizjak M., Maurer M. (2024). New Insights into Chronic Inducible Urticaria. Curr. Allergy Asthma Rep..

[57] Srisuwatchari W., Kanchanaphoomi K., Nawiboonwong J., Thongngarm T., Sompornrattanaphan M. (2023). Food-Dependent Exercise-Induced Anaphylaxis: A Distinct Form of Food Allergy—An Updated Review of Diagnostic Approaches and Treatments. Foods.

[58] Rossi C.M., Lenti M.V., Di Sabatino A. (2022). Adult Anaphylaxis: A State-of-the-Art Review. Eur. J. Intern. Med..

[59] Antolín-Amérigo D., Vidal-Albareda C., De Olano D.G., De La Hoz-Caballer B. (2024). Current Update on Anaphylaxis: Anaphylaxis Management in Recent Guidelines. Eur. Ann. Allergy Clin. Immunol..

[60] Christensen M.J., Eller E., Kjaer H.F., Broesby-Olsen S., Mortz C.G., Bindslev-Jensen C. (2019). Exercise-Induced Anaphylaxis: Causes, Consequences, and Management Recommendations. Expert Rev. Clin. Immunol..

[61] Poziomkowska-Gęsicka I. (2022). Idiopathic Anaphylaxis? Analysis of Data from the Anaphylaxis Registry for West Pomerania Province, Poland. Int. J. Environ. Res. Public. Health.

[62] Tanno L.K., Bierrenbach A.L., Simons F.E.R., Cardona V., Thong B.Y.-H., Molinari N., Calderon M.A., Worm M., Chang Y.-S., On behalf the Joint Allergy Academies (2018). Critical View of Anaphylaxis Epidemiology: Open Questions and New Perspectives. Allergy Asthma Clin. Immunol..

[63] Ansley L., Bonini M., Delgado L., Del Giacco S., Du Toit G., Khaitov M., Kurowski M., Hull J.H., Moreira A., Robson-Ansley P.J. (2015). Pathophysiological Mechanisms of Exercise-induced Anaphylaxis: An EAACI Position Statement. Allergy.

[64] Treudler R. (2024). Emerging and Novel Elicitors of Anaphylaxis: Collegium Internationale Allergologicum Update 2024. Int. Arch. Allergy Immunol..

[65] Wąsik J., Likońska A., Kurowski M. (2024). IgE-Mediated Allergy and Asymptomatic Sensitization to Cannabis Allergens—Review of Current Knowledge and Presentation of Six Cases. Medicina.

[66] Gromek W., Kołdej N., Świtała S., Majsiak E., Kurowski M. (2024). Revisiting Latex-Fruit Syndrome after 30 Years of Research: A Comprehensive Literature Review and Description of Two Cases. J. Clin. Med..

[67] Jankowski W.M., Przychodniak D., Gromek W., Majsiak E., Kurowski M. (2025). Edible Insects as an Alternative Source of Nutrients: Benefits, Risks, and the Future of Entomophagy in Europe—A Narrative Review. Foods.

[68] Jankowski W., Przychodniak D., Kurowski M. (2024). LTP Syndrome and Edible Insect Allergy in a Patient with Recurrent Anaphylaxis Incidents. Pol. J. Allergol..

[69] Mellerowicz E.J., Asady A., Maurer M., Altrichter S. (2019). Angioedema Frequently Occurs in Cholinergic Urticaria. J. Allergy Clin. Immunol. Pract..

[70] Diaz V.L., Gribbons K.B., Yazdi-Nejad K., Kuemmerle-Deschner J., Wanderer A.A., Broderick L., Hoffman H.M. (2023). Cold Urticaria Syndromes: Diagnosis and Management. J. Allergy Clin. Immunol. Pract..

[71] Bizjak M., Rutkowski K., Asero R. (2024). Risk of Anaphylaxis Associated with Cold Urticaria. Curr. Treat. Options Allergy.

[72] Bizjak M., Korošec P., Košnik M., Šelb J., Bidovec-Stojkovič U., Svetina M., Zver S., Dinevski D., Rijavec M. (2025). Cold-Induced Anaphylaxis: New Insights into Clinical and Genetic Characteristics. Front. Immunol..

[73] Rujitharanawong C., Kulthanan K., Tuchinda P., Chularojanamontri L., Metz M., Maurer M. (2022). A Systematic Review of Aquagenic Urticaria—Subgroups and Treatment Options. J. Allergy Clin. Immunol. Pract..

[74] Asady A., Ruft J., Ellrich A., Hawro T., Maurer M., Altrichter S. (2017). Cholinergic Urticaria Patients of Different Age Groups Have Distinct Features. Clin. Exp. Allergy.

[75] Kolkhir P., Metz M., Altrichter S., Maurer M. (2017). Comorbidity of Chronic Spontaneous Urticaria and Autoimmune Thyroid Diseases: A Systematic Review. Allergy.

[76] Kolkhir P., Borzova E., Grattan C., Asero R., Pogorelov D., Maurer M. (2017). Autoimmune Comorbidity in Chronic Spontaneous Urticaria: A Systematic Review. Autoimmun. Rev..

[77] Bizjak M., Košnik M., Dinevski D., Thomsen S.F., Fomina D., Borzova E., Kulthanan K., Meshkova R., Ahsan D.M., Al-Ahmad M. (2022). Risk Factors for Systemic Reactions in Typical Cold Urticaria: Results from the COLD-CE Study. Allergy.

[78] Alrafiaah A.S., Netchiporouk E., Ben-Shoshan M. (2023). Cold-Induced Anaphylaxis Triggered by Drinking Cold Water. Allergol. Immunopathol..

[79] Bizjak M., Košnik M., Dinevski D., Thomsen S.F., Fomina D., Borzova E., Kulthanan K., Meshkova R., Aarestrup F.M., Ahsan D.M. (2022). Adrenaline Autoinjector Is Underprescribed in Typical Cold Urticaria Patients. Allergy.

[80] Bizjak M., Košnik M., Dinevski D., Francis Thomsen S., Fomina D., Borzova E., Kulthanan K., Meshkova R., Aarestrup F., Melina Ahsan D. (2022). Adrenaline Autoinjector Is Under-Prescribed in Typical Cold Urticaria Patients Living in Tropical Climate Countries. Qatar Med. J..

[81] Lelonek E., Matusiak Ł., Wróbel T., Szepietowski J. (2018). Aquagenic Pruritus in Polycythemia Vera: Clinical Characteristics. Acta Derm. Venerol..

[82] Sekar C., Srinivas C., Jacob S. (2011). Aquagenic Pruritus: Beneath Water “Lies”. Indian J. Dermatol..

[83] Fearfield L.A., Gazzard B., Bunker C.B. (1997). Aquagenic Urticaria and Human Immunodeficiency Virus Infection: Treatment with Stanozolol. Br. J. Dermatol..

[84] Parks A., Camisa C. (1986). Aquagenic Angioedema. Cutis.

[85] Kulthanan K., Ungprasert P., Tapechum S., Rujitharanawong C., Kiratiwongwan R., Munprom K., Terhorst-Molawi D., Maurer M. (2021). Vibratory Angioedema Subgroups, Features, and Treatment: Results of a Systematic Review. J. Allergy Clin. Immunol. Pract..

[86] Schubert B., Seitz C.S., Weigel C., Bröcker E.B., Trautmann A. (2007). Angio-Oedema Induced by Bicycling. Br. J. Dermatol..

[87] Patterson R., Mellies C., Blankenship M., Pruzansky J. (1972). Vibratory Angioedema: A Hereditary Type of Physical Hypersensitivity. J. Allergy Clin. Immunol..

[88] Epstein P.A., Kidd K.K., Opitz J.M. (1981). Dermo-distortive Urticaria: An Autosomal Dominant Dermatologic Disorder. Am. J. Med. Genet..

[89] Boyden S.E., Desai A., Cruse G., Young M.L., Bolan H.C., Scott L.M., Eisch A.R., Long R.D., Lee C.-C.R., Satorius C.L. (2016). Vibratory Urticaria Associated with a Missense Variant in *ADGRE2*. N. Engl. J. Med..

[90] Turner P.J., Arasi S., Ballmer-Weber B., Baseggio Conrado A., Deschildre A., Gerdts J., Halken S., Muraro A., Patel N., Van Ree R. (2022). Risk Factors for Severe Reactions in Food Allergy: Rapid Evidence Review with Meta-analysis. Allergy.

[91] Kulthanan K., Ungprasert P., Jirapongsananuruk O., Rujitharanawong C., Munprom K., Trakanwittayarak S., Pochanapan O., Panjapakkul W., Maurer M. (2022). Food-Dependent Exercise-Induced Wheals, Angioedema, and Anaphylaxis: A Systematic Review. J. Allergy Clin. Immunol. Pract..

[92] Leung A.K., Hegde H.R. (1989). Exercise-Induced Angioedema and Asthma. Am. J. Sports Med..

[93] Magen E., Chikovani T. (2019). A Case of Food-Dependent Exercise-Induced Angioedema. J. Allergy Clin. Immunol. Pract..

[94] Caffarelli C., Zinelli C., Trimarco G., Petroccione T., Bernasconi S. (2006). Angio-Oedema in a Child Due to Eating Tomatoes after Exercise. Clin. Exp. Dermatol..

[95] Kobayashi A., Uhara H., Ashida A., Kiniwa Y., Okuyama R. (2011). Thermal Angiooedema Induced by Hot Water. Acta Derm.-Venereol..

[96] Minowa T., Sumikawa Y., Kumagai A., Kamiya T., Uhara H. (2020). Two Cases of Angioedema without Wheals Induced by Exercising or Bathing. Allergol. Int..

[97] Zarnowski J., Treudler R. (2024). Dietary and Physical Trigger Factors in Hereditary Angioedema: Self-Conducted Investigation and Literature Overview. Allergol. Select.

[98] Zotter Z., Csuka D., Szabó E., Czaller I., Nébenführer Z., Temesszentandrási G., Fust G., Varga L., Farkas H. (2014). The Influence of Trigger Factors on Hereditary Angioedema Due to C1-Inhibitor Deficiency. Orphanet J. Rare Dis..

[99] Nygren A., Nordenfelt P., Lindfors A., Mallbris L., Björkander J., Wahlgren C. (2016). Swedish Children with Hereditary Angioedema Report Good Overall Health and Quality of Life despite Symptoms. Acta Paediatr..

[100] Ashrafian H. (2005). Hereditary Angioedema in a Martial Arts Family. Clin. J. Sport Med..

[101] Lamacchia D., Nappi E., Marzio V., Locatelli F., Messina M.R., Heffler E. (2024). Hereditary Angioedema: Current Therapeutic Management and Future Approaches. Curr. Opin. Allergy Clin. Immunol..

[102] Kozłowski M., Żurawska K., Poziomkowska-Gęsicka I. (2024). The Latest Therapeutic Reports on Short-Term and Long-Term Prophylaxis in the Treatment of Hereditary Angioedema. Pol. J. Allergol..

[103] Covella B., Giliberti M., Montinaro A., Rossi L., Montinaro V. (2024). Hereditary Angioedema: Novel Molecules for Treatment of Acute Attacks and Long-Term Prophylaxis. Future Pharmacol..

[104] Fung S. (2025). Garadacimab: First Approval. Drugs.

[105] Craig T.J., Reshef A., Li H.H., Jacobs J.S., Bernstein J.A., Farkas H., Yang W.H., Stroes E.S.G., Ohsawa I., Tachdjian R. (2023). Efficacy and Safety of Garadacimab, a Factor XIIa Inhibitor for Hereditary Angioedema Prevention (VANGUARD): A Global, Multicentre, Randomised, Double-Blind, Placebo-Controlled, Phase 3 Trial. Lancet.

[106] Reshef A., Hsu C., Katelaris C.H., Li P.H., Magerl M., Yamagami K., Guilarte M., Keith P.K., Bernstein J.A., Lawo J. (2025). Long-term Safety and Efficacy of Garadacimab for Preventing Hereditary Angioedema Attacks: Phase 3 Open-label Extension Study. Allergy.

[107] Feener E.P., Davie R.L., Murugesan N., Pethen S.J., Hampton S.L., Smith M.D., Audhya P.K., Yea C.M. (2024). Sebetralstat: A Rapidly Acting Oral Plasma Kallikrein Inhibitor for the On-Demand Treatment of Hereditary Angioedema. Drugs Drug Candidates.

[108] Riedl M.A., Farkas H., Aygören-Pürsün E., Psarros F., Soteres D.F., Staevska M., Cancian M., Hagin D., Honda D., Melamed I. (2024). Oral Sebetralstat for On-Demand Treatment of Hereditary Angioedema Attacks. N. Engl. J. Med..

[109] Jindal A.K., Sil A., Aggarwal R., Vinay K., Bishnoi A., Suri D., Rawat A., Kumaran M.S., Saikia B., Sarkar R. (2023). Management of Hereditary Angioedema in Resource-Constrained Settings: A Consensus Statement from Indian Subcontinent. Asia Pac. Allergy.

[110] Honda D., Li P.H., Jindal A.K., Katelaris C.H., Zhi Y.-X., Thong B.Y.-H., Longhurst H.J. (2024). Uncovering the True Burden of Hereditary Angioedema Due to C1-Inhibitor Deficiency: A Focus on the Asia-Pacific Region. J. Allergy Clin. Immunol..

[111] Maurer M., Abuzakouk M., Al-Ahmad M., Al-Herz W., Alrayes H., Al-Tamemi S., Arnaout R., Binghadeer H., Gutta R., Irani C. (2023). Consensus on Diagnosis and Management of Hereditary Angioedema in the Middle East: A Delphi Initiative. World Allergy Organ. J..

